# Non-uniform susceptibility profiles observed in *Staphylococcus aureus*, *Pseudomonas aeruginosa*, and *Acinetobacter baumannii* toward both front-line and novel cationic biocides with diverse structural compositions

**DOI:** 10.1128/spectrum.01041-26

**Published:** 2026-07-16

**Authors:** Germán G. Vargas-Cuebas, Kevin P. C. Minbiole, William M. Wuest

**Affiliations:** 1 Department of Chemistry, Emory University 1371, Atlanta, Georgia, USA; 2 Department of Chemistry and Biochemistry, Villanova University 8210, Villanova, Pennsylvania, USA; University of Guelph College of Biological Science, Guelph, Ontario, Canada

**Keywords:** efflux, antimicrobial resistance, disinfectant, heteroresistance

## Abstract

Heteroresistance, a phenotype of bacterial subpopulations that display differential susceptibility to antimicrobial agents, is a pressing problem that results in antibiotic treatment failure and potentially in resistance development. However, this phenotype remains largely unexplored in the context of biocides. Here, using population analysis profiling (PAP) assays, we investigated susceptibility patterns in *Staphylococcus aureus*, *Acinetobacter baumannii*, and *Pseudomonas aeruginosa* toward four healthcare-associated commercial cationic biocides (benzalkonium chloride, didecyl dimethyl ammonium chloride, octenidine, and chlorhexidine) and four structurally diverse, non-commercial cationic compounds. These agents exhibited a broad range of antimicrobial activity, with minimal inhibitory concentrations (MICs) for commercial biocides ranging from 1 to 125 µM and for the novel compounds from 0.5 to 8 µM across the species tested. Despite this inhibitory activity, bacterial subpopulations capable of growth at concentrations ≥8-fold higher than the MIC of the dominant population were observed at frequencies of approximately 10⁻⁷ to 10⁻⁶, phenotypically analogous to heteroresistance. Loss of this heteroresistance-like phenotype in efflux-deficient mutants may suggest that efflux systems contribute to this phenotype. Notably, it was observed that a hospital-associated *S. aureus* strain exhibited higher frequencies of these less susceptible bacterial subpopulations compared to community-associated and laboratory strains. Overall, these findings demonstrate that non-uniform susceptibility to cationic biocides, similar to the heteroresistance phenotype observed toward antibiotics, can be detected under defined experimental conditions. Given that in-use biocide concentrations are typically substantially higher than the MICs reported here, the clinical and infection prevention implications of this phenotype remain uncertain and warrant further investigation.

**IMPORTANCE:**

Heteroresistance is a phenotype of growing concern in healthcare settings, contributing to antimicrobial resistance and antibiotic treatment failure. Yet, the development of heteroresistance-like phenotypes toward commercial biocides, prevalent in healthcare settings, remains understudied. Herein, we show that heteroresistance-like phenotypes similar to those observed in response to antibiotic treatment are observed toward both commercial and non-commercial biocides, and these phenotypes warrant future investigation.

## INTRODUCTION

Often described as the “silent pandemic,” antimicrobial resistance (AMR) represents one of the most pervasive global health crises of the 21^st^ century, threatening the longevity of current therapeutics for infectious diseases. The Global Burden of Disease Study of 2021 estimated that by 2050 antibiotic resistance-associated deaths could exceed eight million annually if no mitigation strategies are implemented (1). AMR not only increases the morbidity and mortality associated with infections but also undermines routine medical procedures (e.g., surgery, chemotherapy, and organ transplants) that rely on effective antibiotic prophylaxis (2). Addressing this crisis requires a comprehensive understanding of the factors driving AMR emergence and dissemination.

Cationic biocides are a major but often understudied component of effective infection prevention measures used to curb transmission of pathogens. These agents include widely used antiseptics (e.g., chlorhexidine and octenidine dihydrochloride), as well as other quaternary ammonium compounds (QACs) frequently incorporated into disinfectants and other household products. Their use has expanded substantially in healthcare, community, and industrial settings, raising concerns about their potential role as contributors to AMR (3, 4). Importantly, biocide resistance has been linked to reduced susceptibility to clinically relevant antibiotics, suggesting the possibility of cross-resistance or co-selection of resistance determinants (5–7). While cationic biocides are indispensable for infection prevention and control (IPC), their widespread use and associated antibiotic cross-resistance concerns have bolstered interest in understanding biocide-associated tolerance and resistance phenotypes (8). Notably, when used appropriately, biocides remain a cornerstone of effective IPC strategies by limiting the transmission and dissemination of multidrug-resistant (MDR) pathogens, thereby reducing reliance on antibiotics and indirectly mitigating AMR (9).

Heteroresistance has emerged as a clinically significant resistance phenotype that complicates antimicrobial susceptibility testing and contributes to unexplained antibiotic treatment failure (10), yet its prevalence and relevance outside of antibiotics have remained largely unexplored. Heteroresistance is characterized by the coexistence of bacterial subpopulations within an isogenic bacterial population that exhibit markedly different susceptibility profiles to a given antimicrobial. This phenotype is distinct from persistence, which involves transiently dormant, non-replicating cells rather than actively growing subpopulations (11). In heteroresistance, a fraction of the total population is capable of actively grow at antimicrobial concentrations at least eightfold or higher than that required to inhibit the dominant population. This definition, derived from antibiotic studies, is operational and does not necessarily reflect clinical resistance, particularly for biocides where standardized breakpoints are lacking. Despite recognition of heteroresistance in antibiotic contexts, its occurrence in response to cationic biocides has been minimally explored, with only a single report describing this phenotype in relation to chlorhexidine to date (12). Given the central role of cationic biocides in IPC protocols, and their relationship to antibiotic resistance, this represents a critical knowledge gap limiting our understanding of biocide-associated susceptibility heterogeneity.

Our primary goal was to perform a proof-of-concept investigation to assess whether heteroresistance-like phenotypes are observed in response to a selected group of widely used commercial cationic biocides using population analysis profile (PAP) assays. We expanded our assessment of these phenotypes to include novel, non-commercial cationic biocides and examined the potential contribution of efflux systems to this phenotype. Importantly, this study is limited to a defined set of well-characterized laboratory strains and is not intended to assess prevalence or clinical impact. Yet, our findings provide initial insight into non-uniform susceptibility to cationic biocide under controlled experimental conditions and highlight the need for further investigation using clinical and environmental isolates to evaluate potential implications of this heteroresistance-like phenotype for IPC and AMR surveillance.

## RESULTS

### Selection of commercial and novel cationic biocides

**Fig 1:**
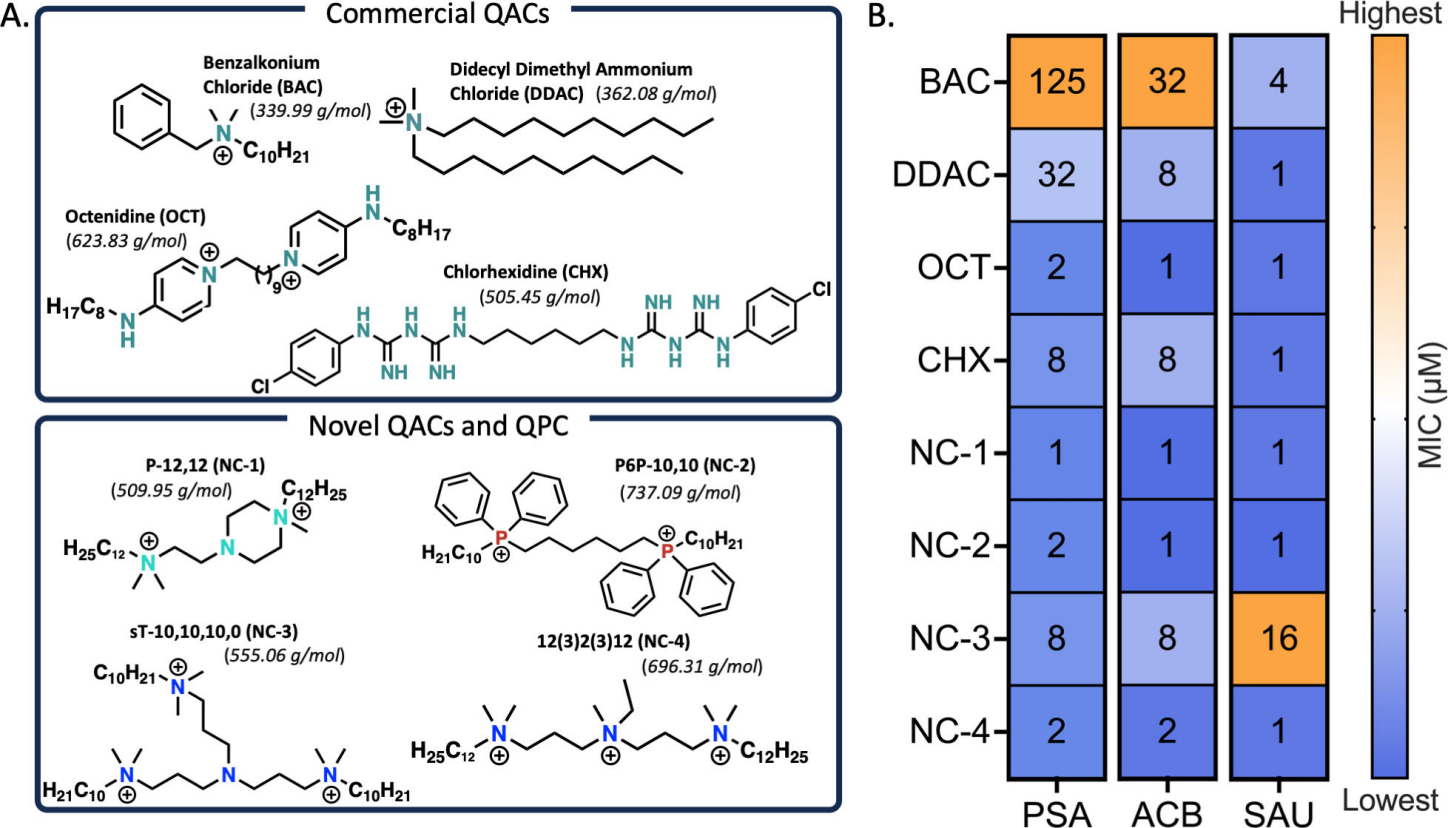
Selection of commercial and novel biocides and baseline antimicrobial activity against *S. aureus, A. baumannii*, and *P. aeruginosa*. (**A**) Chemical structures of selected commercial biocides (top) and novel biocides (bottom). (**B**) Antimicrobial activity measured by minimal inhibitory concentration (MIC) assays of biocides against our three reference strains: *P. aeruginosa* (PSA), *A. baumannii* (ACB), and *S. aureus* (SAU). MIC values provided in µM.

Four commercial biocides and four novel cationic biocides synthesized by our groups were selected to evaluate heteroresistance-like phenotypes toward cationic biocides. The commercial compounds, including benzalkonium chloride (BAC), didecyldimethylammonium chloride (DDAC), octenidine (OCT), and chlorhexidine (CHX), are widely used as antiseptics and disinfectants in healthcare, household, and industrial settings. The two quaternary ammonium compounds (QACs) selected are widely used in a variety of products and observed a significant increase in usage after the COVID-19 pandemic, as most compounds approved for abiotic surface disinfectants contained QACs, of these about a third contained BAC (32%) and about 8% contained the fourth-generation QAC, DDAC (13). On the other hand, OCT and CHX are widely used as antiseptics and with the latter listed in the essential medicine list by the World Health Organization (14, 15). The four novel cationic biocides represent diverse chemical structure and exhibit varying inhibitory and bactericidal activities (Fig. 1A) (16).

The selected biocides show differing mode of action toward gram-negative bacteria, with mono-QACs (e.g., BAC, DDAC) causing indiscriminate membrane disruption while bolaamphiphiles (e.g., OCT, CHX, P6P-10,10) preferentially disrupting the inner membrane (17). Importantly, the novel compounds have not been used in commercial applications (i.e., no previous exposure) and exhibit strong antimicrobial activity in minimal inhibitory concentration (MIC) assays, generally in the single-digit micromolar (µM) range, against the three tested strains: the gram-negatives *P. aeruginosa* (PSA) and *A. baumannii* (ACB) and the gram-positive *S. aureus* (SAU) (Fig. 1B).

### Heteroresistance-like phenotypes observed toward commercial cationic biocides

Population analysis profile (PAP) assays revealed non-uniform susceptibility patterns consistent with heteroresistance-like phenotype to multiple commercial biocides. PAP assays, considered the gold standard for heteroresistance detection, enable the quantification of bacterial subpopulations capable of surviving antimicrobial concentrations higher than those inhibiting the dominant population. Because no standardized resistance break points exist for cationic biocides, three distinct susceptibility profiles were operationally defined as follows: growth across all concentration tested (operationally defined as *resistant*); growth of a subpopulation at a concentration ≥8-fold than that inhibiting the dominant population (operationally defined as *heteroresistant*); or complete clearance of the population within an 8-fold concentration range (operationally defined as *susceptible*) (18).

**Fig 2:**
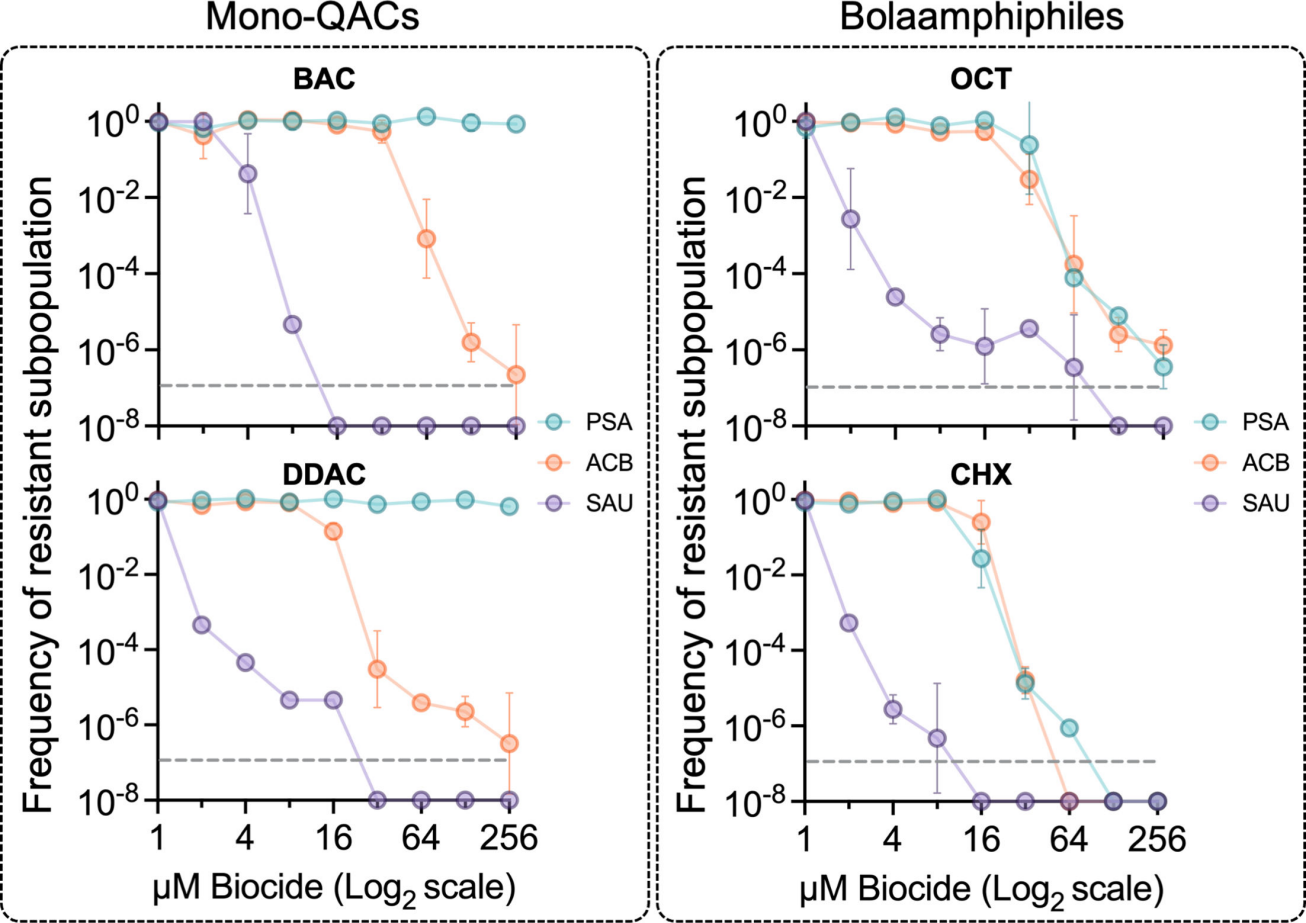
Bacteria display heteroresistance to commonly used commercial disinfectants and antiseptics. Heteroresistance exhibited by *P. aeruginosa* (PSA), *A. baumannii* (ACB), and *S. aureus* (SAU) toward mono-QACs BAC and DDAC (left panel) and bolaamphiphiles OCT and CHX (right panel).

All three susceptibility profiles were observed with the tested commercial biocides (Fig. 2). *P. aeruginosa* exhibited growth across the tested concentration range for BAC and DDAC (BAC, 0–512 µM or 0–174.07 µg/mL; DDAC, 0–256 µM or 0–92.7 µg/mL), displayed heteroresistance-like patterns to OCT, and remained susceptible to CHX. *A. baumannii* displayed heteroresistance-like phenotype to BAC, DDAC, and OCT but was susceptible to CHX. These observations were consistent with our prior findings of trailing growth phenotypes during MIC testing of biocides against *A. baumannii* (19). The *S. aureus* strain also exhibited heteroresistance-like profiles to three of the four commercial biocides (BAC, DDAC, and OCT) although the concentrations tolerated were generally lower than those observed with the gram-negative species.

Because antimicrobials susceptibility can be influenced by bacterial density (i.e., inoculum effect) (20), PAP assays were repeated using increased plating volumes to reduce the density of the sample plated. Comparable heteroresistance profiles were observed, indicating that the observed non-uniform susceptibility patterns were not dependent on plating volume under the conditions tested.

### Novel cationic biocides display heteroresistance-like patterns across bacterial species

**Fig 3:**
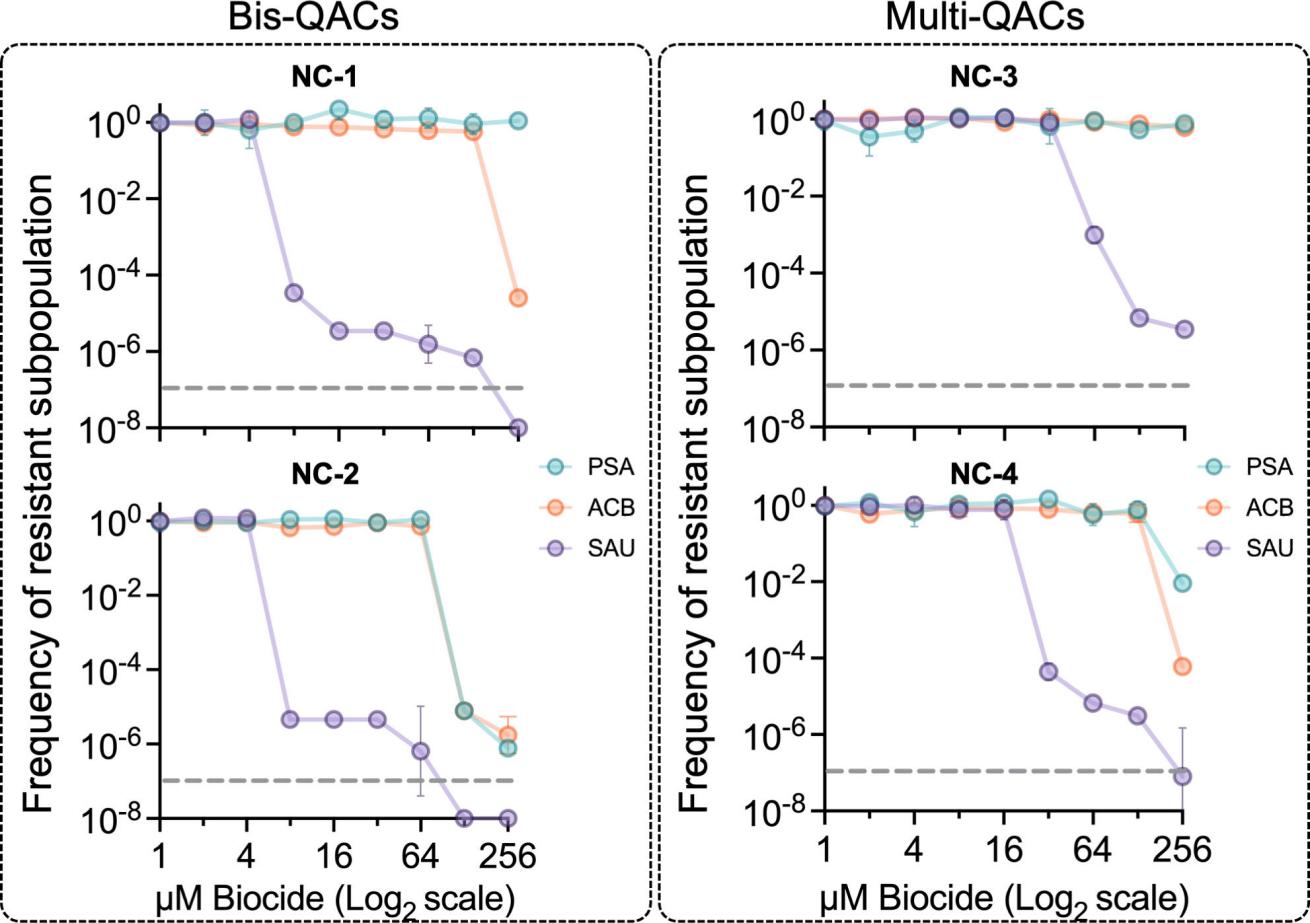
Heteroresistance observed toward novel, chemically diverse cationic biocides. Heteroresistance exhibited by *P. aeruginosa* (PSA), *A. baumannii* (ACB), and *S. aureus* (SAU) toward four chemically diverse novel biocides: the bis-QAC P-12,12 (NC-1) and the bis-QPC P6P-10,10 (NC-2) (left panel) and the multi-QACs sT-10,10,10,0 (NC-3) and 12(3)2 (3)12 (NC-4) (right panel).

Given the consistent detection of heteroresistance-like susceptibility patterns with commercial biocides, we next evaluated heteroresistance using four novel, chemically diverse cationic biocides. The selected compounds include the double-headed QAC (bis-QAC) P-12,12 (NC-1) and the phosphonium-based (bis-QPC) P6P-10,10 (NC-2). In addition, multi-QACs (compounds with three positively charged heads) were selected, including the branched QACs sT-10,10,10,0 (NC-3) and 12(3)2 (3)12 (NC-4) (Fig. 3).

Despite the substantial chemical diversity among these compounds, all three tested strains exhibited either growth across the tested concentration range (0–256 µM) or heteroresistance-like profiles toward the four novel cationic biocides. As observed with commercial biocides, gram-negative strains displayed higher apparent tolerance levels than *S. aureus*. However, differences in susceptibility revealed by PAP assays between *P. aeruginosa* and *A. baumannii* were less pronounced for the novel biocides than for the commercial biocides.

### Frequency, magnitude, and stability of heteroresistance-like phenotypes

**Fig 4:**
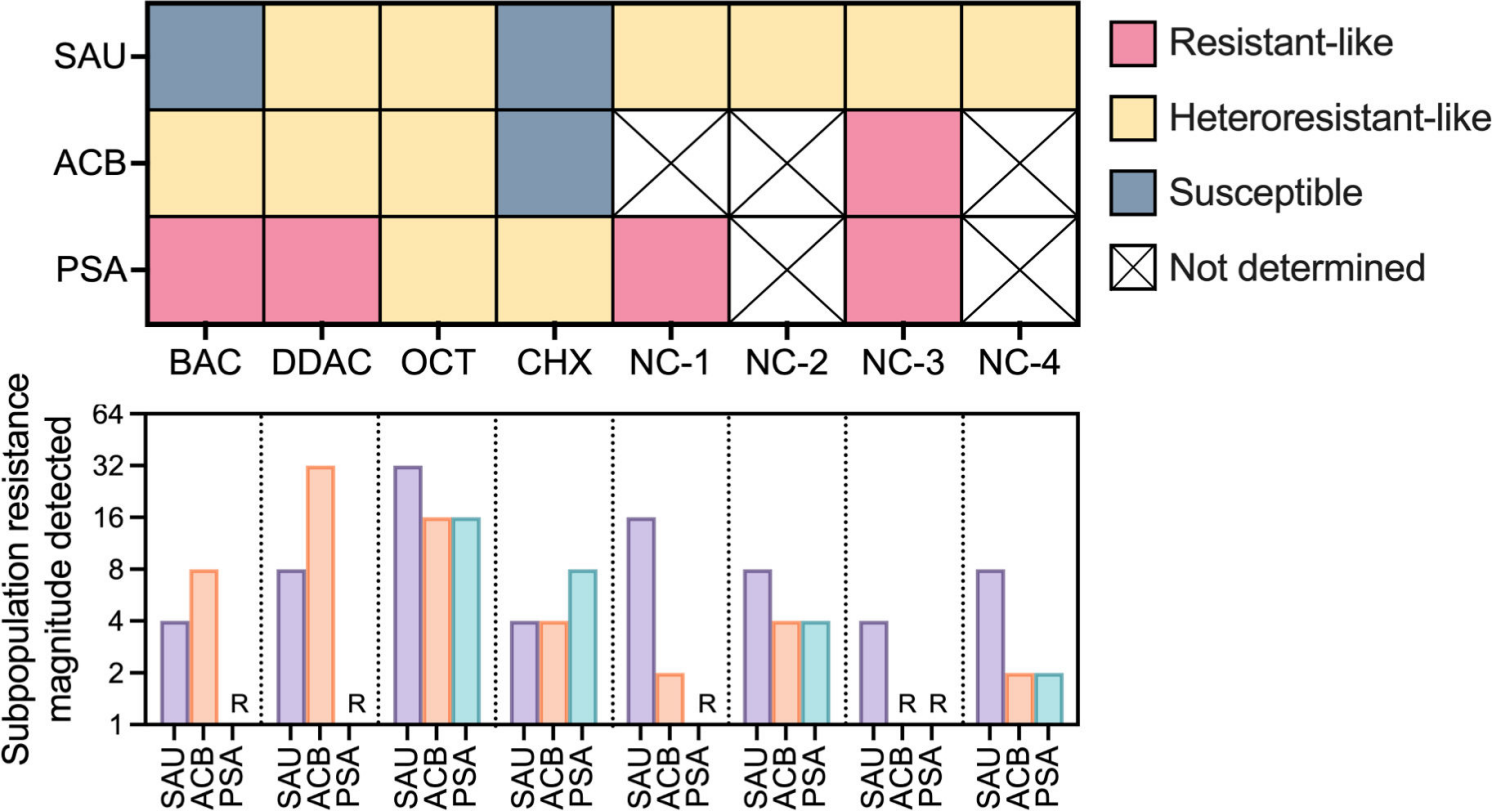
Detection and magnitude of heteroresistance across biocides and species. Summary of resistance phenotypes exhibited by *P. aeruginosa* (PSA), *A. baumannii* (ACB), and *S. aureus* (SAU) to cationic biocides determined by PAP assays (top panel). The resistance magnitude difference between heteroresistant subpopulations observed was plotted (log_2_ scale, bottom panel). BAC, benzalkonium chloride; DDAC, didecyldimethylammonium chloride; OCT, octenidine chloride; CHX, chlorhexidine; NC-1, P-12,12; NC-2, P6P-10,10; NC-3, sT-10,10,10,0; and NC-4, 12(3)2 (3)12.

Several parameters are used to define heteroresistance, including population clonality, the magnitude or level of reduced susceptibility in the bacterial subpopulation, and the stability of this phenotype (11). All experiments were performed using freshly isolated colonies derived from reference bacterial stocks, supporting the assumption of populations clonality. Growth across all tested concentrations was most frequently observed in *P. aeruginosa*, while heteroresistance-like phenotypes were more frequently observed in *S. aureus* (Fig. 4). In several cases, the range of concentration used was not sufficient to fully characterized the phenotype, and these instances are noted accordingly.

The magnitude of reduced susceptibility ranged from 8- to 32-fold higher relative to the maximum concentration tolerated by the dominant population (Fig. 4). The frequency of the subpopulations displaying growth at elevated concentrations consistently ranged from 10^−6^ to 10^−7^. These phenotypes were unstable, as MIC values obtained before and after biocide-free passaging were nearly identical, consistent with previously described heteroresistance behavior rather than stable resistance acquisition or selection.

### Hospital-associated *S. aureus* strain exhibits elevated heteroresistance-like susceptibility profile

**Fig 5:**
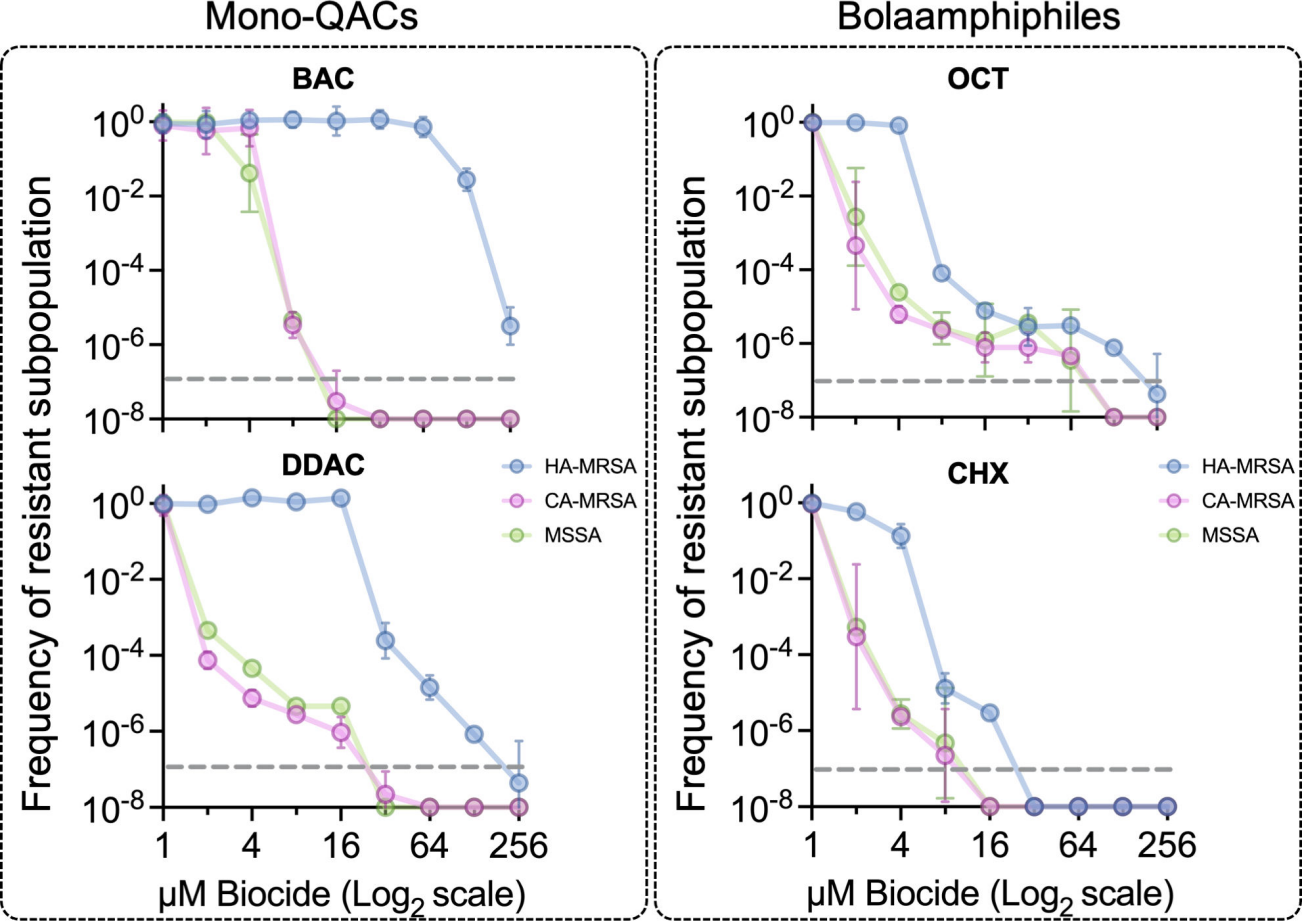
Hospital-derived strain of *S. aureus* displays higher levels of heteroresistance to biocides. Heteroresistance as determined by PAP assays for quaternary ammonium compounds BAC (top left) and DDAC (top right), and antiseptics OCT (bottom left) and CHX (bottom right) using methicillin-susceptible *S. aureus* SH1000 (MSSA), community-acquired methicillin-resistant *S. aureus* USA300-0114 (CA-MRSA), and hospital-acquired methicillin-resistant *S. aureus* ATCC 33,591 (HA-MRSA).

Given that non-uniform susceptibility patterns were observed across both commercial and novel biocides, we considered whether generalized antimicrobial resistance mechanisms (e.g., altered permeability or efflux activity) might contribute to this phenotype. We, therefore, compared heteroresistance-like profiles among three *S*. *aureus* strains: a hospital-associated (HA-MRSA), a community-associated (CA-MRSA), and the MSSA laboratory strain SH1000 (Fig. 5).

The HA-MRSA isolate exhibited higher frequencies of subpopulations capable of growing at elevated biocide concentrations compared to the CA-MRSA and MSSA strains. This observation is presented descriptively, and while it may reflect differences in antimicrobial exposure history or efflux activity differences, no causal relationship can be inferred from these data alone. Further exploration is needed to identify the underlying mechanism(s) for these differences observed and to determine if these are related to efflux activity differences between these strains (21, 22).

### Efflux pump deficiency is associated with loss of detectable heteroresistance-like phenotype

**Fig 6:**
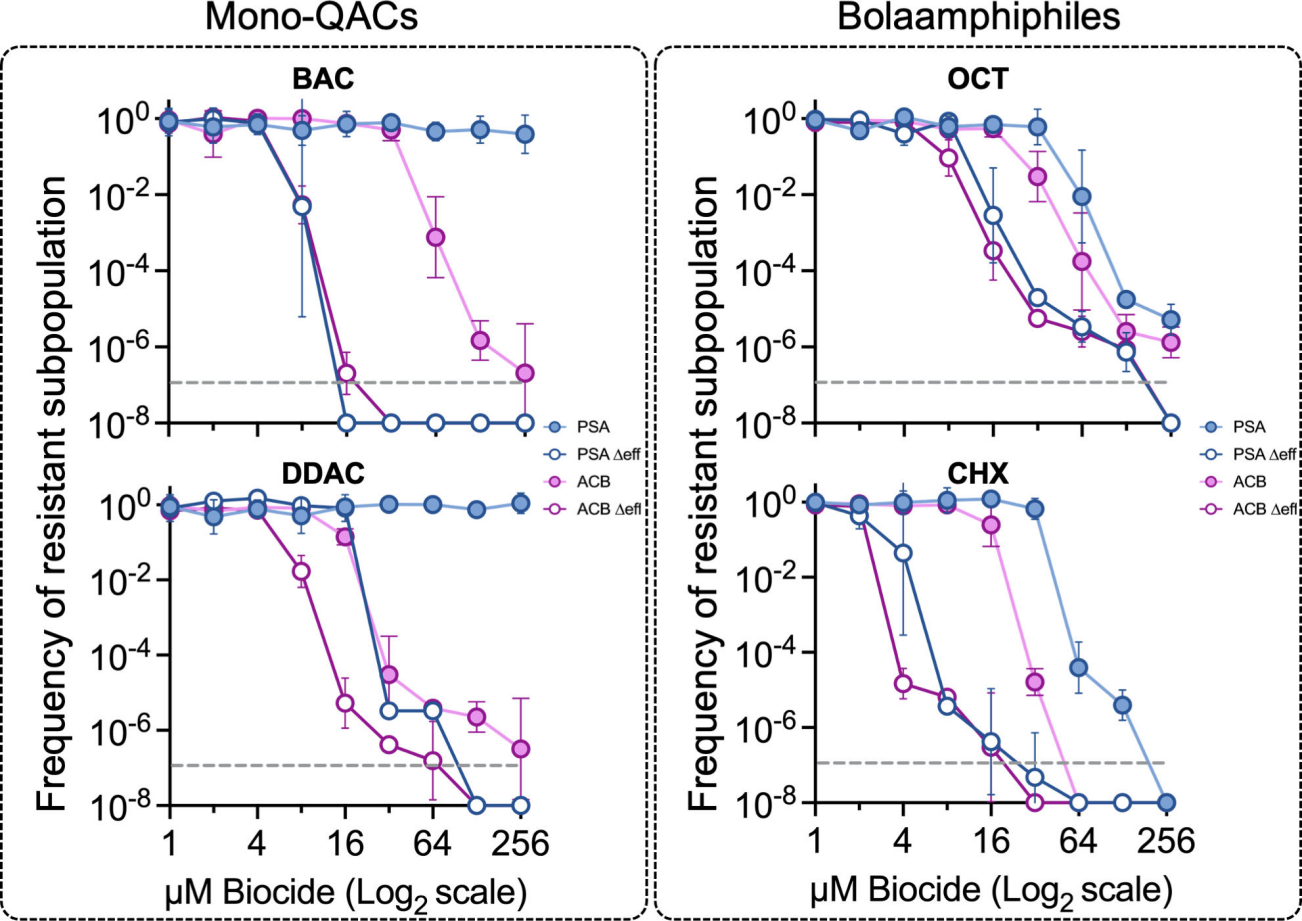
Efflux activity contributes to cationic biocides heteroresistance. Heteroresistance as determined by PAP assays for quaternary ammonium compounds BAC (top left) and DDAC (top right) and antiseptics OCT (bottom left) and CHX (bottom right) in efflux-deficient strains and the isogenic wild-type parental strains of *A. baumannii* and *P. aeruginosa*.

To evaluate the potential contribution of efflux systems in the heteroresistance-like susceptibility patterns observed, we examined this phenotype using efflux-deficient *A. baumannii* and *P. aeruginosa* mutants, alongside their isogenic parental strains (Table 1). Compared to their parental strains, efflux-deficient mutants did not exhibit detectable heteroresistance-like profiles to any biocides under the conditions tested (Fig. 6).

**TABLE 1:** Strains used in this study and pertinent information

Strain	Strain details	Source or reference
*S. aureus* SH1000	Wild-type laboratory strain	(23)
*A. baumannii* ATCC17978	Wild-type laboratory strain	(24)
*P. aeruginosa* (PAO1)	Wild-type laboratory strain	(25)
*S. aureus* USA300-0114	Community-acquired methicillin-resistant *S. aureus* (CA-MRSA)	(26)
*S. aureus* ATCC 33,591	Hospital-acquired methicillin-resistant *S. aureus* (HA-MRSA)	(27)
*A. baumannii ∆ efflux*	17,978 ΔadeB ΔadeJ *surA::Tn26*	Phillip Rather, PhD
*P. aeruginosa* PA14	PA14 wild-type strain	Genentech
*P. aeruginosa ∆ efflux*	PA14 efflux-deficient strain (∆*mexXY, ∆mexCD-oprJ, ∆mexJK-opmH, ∆mexEF-oprN, ∆oprD, ∆mexGHI-opmD, ∆mexAB*)	Genentech

These findings suggest that efflux systems may contribute to the observed phenotype. However, given the genetic complexity of the mutants used and potential pleiotropic effects (e.g., altered membrane integrity), the role of other systems other than efflux activity cannot be excluded.

## DISCUSSION

Biocides are a cornerstone of infection prevention and control protocols that have served for decades as a first line of defense against pathogen transmission (28, 29). Yet, studies exploring resistance phenotypes toward this important class of antimicrobials remain scarce. Owing to their broad antimicrobial activity, these compounds are routinely used in healthcare settings to reduce microbial burden on both biotic and abiotic surfaces. This role is critical to curb infections caused by major nosocomial pathogens, including *S. aureus*, *A. baumannii,* and *P. aeruginosa* (30).

Antibiotic heteroresistance has gained increasing recognition due to its association with antibiotic therapeutics failure mediated by resistant bacterial subpopulations (10, 31). In contrast, biocide-associated heteroresistance-like susceptibility profiles have remained largely unexplored, in part, due to the absence of standardized susceptibility breakpoints. In this study, we used phenotypically derived susceptibility profiles to examine heteroresistance-like susceptibility patterns across a selection of widely used commercial biocides and structurally distinct, non-commercial cationic biocides using PAP assays (Fig. 1). Our findings demonstrate that non-uniform susceptibility patterns, consistent with those observed in heteroresistance, can be detected under defined experimental conditions across multiple cationic biocides and several bacterial species (Fig. 2 and 3). Importantly, these observations are limited to the strains tested and do not imply prevalence of generalizability.

Although heteroresistance-like patterns were observed less frequently with CHX, *P. aeruginosa* displayed non-uniform susceptibility profiles to CHX and OCT and exhibited growth across tested concentrations for BAC and DDAC. While the highest concentration tested in our experimental setup is orders of magnitude below of those used generally for disinfection, these concentrations closely align with those use in other products or processes, such as mosquitocide products, sanitation, and food processing, ranging from 0.1% to 0.2%, and well within the concentration range for BAC used as a preservative of ophthalmic drops, ranging from 0.004% to 0.02% (32, 33). These patterns are consistent with the known contribution of intrinsic resistance features of *P. aeruginosa,* including low outer membrane permeability and efflux activity (34). The increased activity of OCT and CHX against *P. aeruginosa* may reflect their bolaamphiphilic structures and preferential inner membrane targeting, distinguishing their mechanism of action from that of mono-QACs BAC and DDAC (17).

We also observed differences in susceptibility patterns between commercial and non-commercial compounds, with the latter often exhibiting a higher propensity to tolerance phenotypes displayed by the strains under the conditions tested. These novel cationic biocides: P-12,12 (NC-1), 12(3)2 (3)12 (NC-2), sT-10,10,10,0 (NC-3), and P6P-10,10 (NC-4), span a range of chemical structures, including bis- and multi-QACs. While these observations may suggest that physicochemical properties influence susceptibility profiles, the underlying mechanisms for the observed phenotypes remain to be determined. The observed patterns are consistent with generalized mechanisms that reduce the effective biocide concentrations, such as decrease permeability or efflux; however, these interpretations remain speculative and require additional experimentation for direct validation (35).

To further explore potential contributors to these non-uniform susceptibility profiles, we compared strains associated with hospital, community, and laboratory settings. The hospital-associated MRSA isolate exhibited higher frequencies of subpopulations capable of growing at elevated biocide concentrations compared to the community-associated MRSA and MSSA strain (Fig. 5). Nonetheless, this observation remains descriptive, and while it may reflect differences in antimicrobial exposure history, no causal relationship between clinical environment and heteroresistance-like phenotypes can be established from these data at this point. However, further exploration of the differences between these strains might shed light into differences in biocide tolerance and the phenotypes that allow specific hospital-adapted strains to survive within these environments that experience heightened disinfection protocols.

Similarly, experiments performed using efflux-deficient *A. baumannii* and *P. aeruginosa* strains showed loss of detectable heteroresistance-like susceptibility profiles under the tested conditions relative to their isogenic parental strains (Fig. 6). These findings suggest that efflux systems may contribute to the observed phenotype; yet, given the complexity of the mutants used and the potential for pleiotropic effects (including altered membrane integrity or overall fitness), efflux cannot be considered the sole determinant behind the phenotype observed. Additional mechanisms, including membrane adaptation or stress response pathways, are likely involved and warrant further investigation.

We acknowledge several limitations of this study. First, many of the strains used are long-standing laboratory reference strains that, while ideal for laboratory experimentation, do not capture the genetic and phenotypic diversity of the contemporary clinical isolates population. While these strains provide a controlled framework for detecting and characterizing heteroresistance-like phenotypes, they are not suitable for assessing overall prevalence or epidemiological relevance. Future studies incorporating larger panels of recent clinical and environmental isolates will be necessary to evaluate the distribution and variability of biocide-related susceptibility phenotypes (Fig. 4). Second, the absence of established resistance breakpoints for biocides limits direct clinical interpretation of PAP assay results. Additionally, the concentrations tested in this study are substantially lower than typical in-use concentrations for many biocides, which are often used at concentrations orders of magnitude higher, particularly for disinfection. Therefore, the extent to which these findings translate to reduced efficacy under real-world conditions remains unclear. Defining biocide susceptibility thresholds and incorporating application-relevant parameters (e.g., formulation, contact time, surface interactions) will be critical next steps to consider. Third, we note that the operational definition of heteroresistance used here (≥8-fold difference to the dominant population) is derived from antibiotic literature and does not directly translate to biocide activity. This highlights the need for a standardized framework to define and interpret biocide susceptibility heterogeneity. Finally, we acknowledge that the use of mutants with complex genotypes limits the potential conclusions that can be drawn from the experiments performed due to possible pleiotropic effect of the mutations; however, these results raise interesting questions about the overall role of efflux systems in biocide susceptibility profiles. In addition, other mechanisms (e.g., membrane permeability) could contribute to the observed results.

In summary, this study provides an initial, controlled assessment of non-uniform susceptibility responses to structurally and functionally diverse cationic biocides across several bacterial species. Our findings suggest that multiple factors, including intrinsic resistance mechanisms and the physicochemical properties of the compounds tested, may influence these phenotypes. However, given the limitations outlined above, these results should be interpreted as hypothesis-generating rather than definitive evidence of widespread or clinically impactful heteroresistance-like phenotypes. Further investigation is required to determine the biological and practical significance of these observations in the context of infection prevention and antimicrobial resistance.

## MATERIALS AND METHODS

### Bacterial strains and growth conditions

Strains used in this study are listed in Table 1. Strains of *S. aureus*, *A. baumannii,* and *P. aeruginosa* were streaked on fresh Mueller-Hinton broth (MHB) agar plates and grown overnight (16–20 h) at 37°C. Single isolated colonies were used to inoculate liquid MHB cultures grown with shaking (200 rpm) at 37°C overnight (16–20 h). For experiments involving hospital-acquired MRSA strain *S. aureus* (ATCC 33591), cultures were grown in Tryptic soy broth (TSA) to maintain consistency with recommended growth conditions for this isolate. Optical density measurements were obtained at 600 nanometers (OD_600_) using a SpectraMax iD3 plate reader (Molecular Devices, United States). The efflux-deficient mutants used in this study have been previously characterized; however, the presence of multiple deletions and corresponding potential pleiotropic effects (e.g., altered membrane integrity or fitness defects) cannot be excluded.

### Cationic biocides used in the study

Eight different chemically diverse cationic biocides were used in the study (Table 2). Representative commercial compounds, benzalkonium chloride (BAC), didecyldimethylammonium chloride (DDAC), octenidine (OCT), and chlorhexidine (CHX) were purchased (TCI America). The novel compounds P-12,12 (NC-1), P6P-10,10 (NC-2), sT-10,10,10,0 (NC-3), and 12(3)2(3)12 (NC-4) were also synthesized in house, as previously described (33, 36, 37).

**TABLE 2:** Cationic biocides used in this study

Compound	Abbreviation	Molecular weight	Compound details
Benzalkonium chloride	BAC	339.99	Mono-QAC
Didecyldimethylammonium chloride	DDAC	368.04	Mono-QAC
Octenidine	OCT	623.83	Bolaamphiphile
Chlorhexidine	CHX	505.45	Bolaamphiphile
P-12,12	NC-1	509.95	Bis-QAC
P6P-10,10	NC-2	737.09	Bis-QPC
sT-10,10,10,0	NC-3	555.06	Multi-QAC
12(3)2(3)12	NC-4	696.31	Multi-QAC

### Minimal inhibitory concentration assays

Biocides were serially diluted twofold in 96-well plates from stock solutions prepared at a concentration of 1.0 mM, yielding 11 test concentrations. These stock solutions were prepared in 10% DMSO that, after serial dilutions, the working DMSO concentration ranged from 2.5% to 0.001% in the plate. Solvent controls were included in each plate accordingly, and no growth inhibition was observed within this solvent range. Positive growth and negative media controls were included in each plate for all strains.

Overnight cultures (16–20 h) of *S. aureus* (SH1000), *A. baumannii* (ATCC17978), and *P. aeruginosa* (PAO1) were diluted to ~10^6^ colony-forming units per milliliter (CFU/mL) in MHB, regrown to mid-exponential phase (~4–6 h) to obtain actively growing cells, and then diluted again to ~10^6^ CFU/mL to serve as the assay inoculum. A total of 100 μL of bacterial suspension was mixed with 100 µL of compound solution previously prepared a in U-bottom 96-well plate (Corning, 351177), resulting in a final inoculum of ~5 × 10^5^ CFU/mL per well.

Plates were incubated statically at 37°C and monitored daily for 72 h; this extended incubation to capture potential delayed inhibitory growth effects associated with cationic biocides, after which wells were visually evaluated for visible growth. All experiments indicated general concordance between between 24-h and 72-h MIC readings; however, the 72-h incubation timepoint was used to ensure the detection of late any late-emerging growth. The MIC was defined as the lowest concentration of the compound resulting in no visible bacterial growth to the naked eye based on the highest MIC value in three independent biological experiments.

### Population analysis profile assays

Solid MHB plates were prepared using eight increasing concentrations for each biocide (2 µM–256 µM, except BAC with highest 512 µM), including MHB-only control plates prepared from the same media batch. Bacterial strains were grown overnight (16–24 h) in their respective media from isolated ƒ colonies. These cultures were then used to prepare 10-fold dilutions the next day (dilutions ranging from undiluted to 10^−6^).

Aliquots (5 µL) of each dilution were carefully spotted onto pre-dried biocide-containing plates and corresponding no-biocide control plates. Spots were allowed to air-dry in a biosafety cabinet for approximately 15–30 min before incubation at 37°C for 18–24 h. This incubation period was selected to prevent overgrowth of colonies and allow accurate enumeration of CFUs.

CFU/mL were calculated for each condition by accounting for dilution factors and plated volume. Growth at each biocide concentration was normalized to the no-biocide control plates to determine log reductions at the different biocide concentrations. Average values were obtained from three independent biological replicates.

Heteroresistance-like profiles were operationally defined as the presence of bacterial subpopulations capable of growth at concentrations ≥8-fold higher than the concentration inhibiting growth of the dominant population. This definition, adapted from antibiotic literature, is used here for comparative purposes only given the lack of standardized breakpoints for biocides and is not intended to reflect clinical or disinfection efficacy effects of the biocides tested.

## Data Availability

All data supporting the findings of this study are available within the article and Materials and Methods section. Additional raw data files are available from the corresponding author upon reasonable request.
